# Quantitative assessment of renal structural and functional changes in chronic kidney disease using multi-parametric magnetic resonance imaging

**DOI:** 10.1093/ndt/gfz129

**Published:** 2019-06-29

**Authors:** Charlotte E Buchanan, Huda Mahmoud, Eleanor F Cox, Thomas McCulloch, Benjamin L Prestwich, Maarten W Taal, Nicholas M Selby, Susan T Francis

**Affiliations:** 1 Sir Peter Mansfield Imaging Centre, School of Physics and Astronomy, University of Nottingham, Nottingham, UK; 2 Centre for Kidney Research and Innovation, University of Nottingham, Royal Derby Hospital Campus, Nottingham, UK; 3 Nottingham University Hospitals NHS Trust, Nottingham, UK

**Keywords:** chronic kidney disease, haemodynamics, magnetic resonance imaging, multi-parametric, renal function

## Abstract

**Background:**

Multi-parametric magnetic resonance imaging (MRI) provides the potential for a more comprehensive non-invasive assessment of organ structure and function than individual MRI measures, but has not previously been comprehensively evaluated in chronic kidney disease (CKD).

**Methods:**

We performed multi-parametric renal MRI in persons with CKD (*n* = 22, 61 ± 24 years) who had a renal biopsy and measured glomerular filtration rate (mGFR), and matched healthy volunteers (HV) (*n* = 22, 61 ± 25 years). Longitudinal relaxation time (*T*_1_), diffusion-weighted imaging, renal blood flow (phase contrast MRI), cortical perfusion (arterial spin labelling) and blood-oxygen-level-dependent relaxation rate (*R*_2_*) were evaluated.

**Results:**

MRI evidenced excellent reproducibility in CKD (coefficient of variation <10%). Significant differences between CKD and HVs included cortical and corticomedullary difference (CMD) in *T*_1_, cortical and medullary apparent diffusion coefficient (ADC), renal artery blood flow and cortical perfusion. MRI measures correlated with kidney function in a combined CKD and HV analysis: estimated GFR correlated with cortical *T*_1_ (*r* = −0.68), *T*_1_ CMD (*r* = −0.62), cortical (*r* = 0.54) and medullary ADC (*r* = 0.49), renal artery flow (*r* = 0.78) and cortical perfusion (*r* = 0.81); log urine protein to creatinine ratio (UPCR) correlated with cortical *T*_1_ (*r* = 0.61), *T*_1_ CMD (*r* = 0.61), cortical (*r* = −0.45) and medullary ADC (*r* = −0.49), renal artery flow (*r* = −0.72) and cortical perfusion (*r* = −0.58). MRI measures (cortical *T*_1_ and ADC, *T*_1_ and ADC CMD, cortical perfusion) differed between low/high interstitial fibrosis groups at 30–40% fibrosis threshold.

**Conclusion:**

Comprehensive multi-parametric MRI is reproducible and correlates well with available measures of renal function and pathology. Larger longitudinal studies are warranted to evaluate its potential to stratify prognosis and response to therapy in CKD.

## INTRODUCTION

Chronic kidney disease (CKD) is a major global health burden [1] affecting 13% of adults [2], with rates predicted to rise by 5–8% per annum [3]. CKD encompasses a range of aetiologies but progresses by a combination of common pathological mechanisms including glomerular capillary hypertension and hyperfiltration, inflammation, vascular rarefaction, hypoxia and fibrosis [4, 5]. Advanced magnetic resonance imaging (MRI) techniques offer the potential to assess and quantify pathophysiological processes in the kidneys non-invasively (as opposed to renal biopsy) and without the use of gadolinium contrast agents. However, MRI techniques are underutilized in nephrology, in part as they have been applied in isolation. Conceptually, a more comprehensive assessment of renal structure, microstructure, haemodynamics and oxygenation is provided by multi-parametric MRI. The value of multi-parametric MRI has been described in cancer [6, 7], for example, in guiding prostate biopsies, where it has helped to reduce the biopsy rate [8, 9], and in animal models of CKD [10]. To date, multi-parametric MRI has not been applied to patients with CKD. 

Here, we assess the use of multi-parametric MRI in persons with CKD and healthy volunteers (HVs), acquiring diffusion weighted imaging (DWI) [11] and *T*_1_ [12] (spin-lattice relaxation time) mapping measures to study renal microstructure, phase contrast MRI (PC-MRI) and Arterial Spin Labelling (ASL) [13] to study renal haemodynamics and blood-oxygen-level-dependent (BOLD) [14] transverse relaxation rate (*R*_2_*) to assess renal tissue oxygenation [15]. We report the reproducibility of each MRI measure, and compare MRI measures with biochemical measures of kidney function and histopathology.

## MATERIALS AND METHODS

### Participants

Over a 12 month period, persons with CKD Category G3–4 [estimated glomerular filtration rate (eGFR) 15–59 mL/min/1.73 m^2^] aged ≥18 years who had undergone renal biopsy as part of clinical care within the previous 90 days were recruited. The decision to perform a kidney biopsy was based on individualized clinical decision-making by a senior nephrologist; our centre performs ∼130 native renal biopsies per annum. Exclusion criteria were contraindications to MRI, an episode of acute kidney injury within the preceding 3 months, renal transplant recipients, known iodine allergy, pregnancy and inability to provide fully informed consent. A HV group (eGFR >60 mL/min/1.73 m^2^ and no proteinuria) who matched 1:1 to each CKD patient for age (±5 years) and gender was recruited.

The study was approved by the East Midlands Research Ethics committee and registered at ClinicalTrials.gov (Identifier: NCT03578523). All participants gave written informed consent.

### Clinical assessment

Demographic data, medical history and anthropomorphic measurements were collected at study entry. This included blood pressure, eGFR from serum creatinine concentration using the Chronic Kidney Disease Epidemiology Collaboration (CKD-EPI) formula [16] and urine protein to creatinine ratio (UPCR) measured from a single early morning urine sample.

In the CKD group only, measured GFR (mGFR) was assessed using iohexol clearance. A total of 5 mL of iohexol (Omnipaque240) was administered intravenously. Blood was sampled at 120, 180 and 240 min in patients with eGFR >40 mL/min/1.73 m^2^, with a further sample at 360 min in patients with eGFR <40 mL/min/1.73 m^2^. Samples were centrifuged and serum frozen at −80°C until single batch analysis. Iohexol clearance was calculated from the rate of decline in iohexol concentration corrected for body surface area (BSA), measured using reverse-phase high performance liquid chromatography (Chemical Pathology Laboratory, John Radcliffe Hospital, Oxford, UK).

### Multi-parametric renal MRI

The multi-parametric renal MRI scan protocol follows that described in Cox *et al.* [17]. This was performed in the CKD group within 7 days of biochemical measures, and again after 7–14 days to assess repeatability. HVs had a single scan.

Scanning was performed on a 3T Philips Ingenia scanner (Multi-Transmit, d-Stream). Balanced turbo field echo (bTFE) localizer scans were acquired in three orthogonal planes to quantify kidney volume and plan placement of the five contiguous coronal oblique slices collected for multi-parametric MRI (288 mm × 288 mm field-of-view with 3 mm × 3 mm in-plane resolution, 5 mm slice thickness). Data were acquired at end-expiration.

#### T_1_ mapping

A respiratory-triggered inversion recovery (IR) sequence [inversion times (TIs): 200/300/400/500/600/700/800/900/1000/1100/1200/1300/1500 ms, temporal slice spacing 58 ms] with fat-suppressed spin-echo EPI (SE-EPI) readout (SENSE 2.3/TE 27 ms) was collected. In addition, a 1.5 mm × 1.5 mm × 5 mm IR balanced fast field echo (IR-bFFE) *T*_1_ dataset was acquired at the same TIs (temporal slice spacing 450 ms) in both ascend and descend slice order.

#### DWI

Respiratory-triggered fat-suppressed SE-EPI DWI data (SENSE 2.3/TE 67 ms) was acquired at 11 *b*-values (0/5/10/20/30/50/100/200/300/400/500 s/mm^2^) in three orthogonal directions to reduce the influence of diffusion anisotropy. A maximum *b*-value of 500 s/mm^2^ was chosen due to the limitation of the 3T Ingenia gradients in reaching a higher *b*-value while maintaining an acceptable echo time.

#### ASL perfusion

Respiratory-triggered Flow Alternating Inversion Recovery ASL data were acquired with in-plane pre- and post-saturation pulses, a post-label delay (PLD) of 1800 ms and selective (S)/non-selective (NS) thickness of 45/400 mm, with 25 S/NS pairs. Data were collected with a SE-EPI readout (SENSE 2.3/TE 27 ms). An inflow scan (4 S/NS pairs at PLDs 300/500/700/900 ms) and M_0_ scan were acquired for quantification.

#### Renal artery blood flow

A non-contrast enhanced MR angiogram was acquired to plan PC-MRI slice placement prior to bifurcations of the renal artery. PC-MRI data were collected on each renal artery in a breath hold [flip-angle 25°, resolution 1.2 mm × 1.2 mm × 6 mm, 20 phases, velocity encoding (v_ENC_) 100 cm/s].

#### BOLD data

BOLD *R*_2_* data were acquired using a multi-echo fast-field-echo (mFFE) scheme (12 echoes, TE/ΔTE 5/3 ms, SENSE 2, 25° flip angle, three breath-holds).

### Data analysis

#### Multi-parametric renal MRI

##### Total kidney volume

Kidney volume was computed by manually tracing the kidney on the coronal bTFE localizer images (Analyze9^®^, AnalyzeDirect, Overland Park, KS, USA).

##### Renal artery blood flow and global perfusion

Renal artery blood flow was assessed using Q-flow software (Philips Medical Systems, Best, The Netherlands). Mean flow velocity (cm/s), cross-sectional area of the lumen (mm^2^) and bulk renal blood flow (mL/s) over the cardiac cycle were calculated for each renal artery and summed to determine total renal artery flow (mL/min). Global perfusion was calculated for each kidney by correcting renal artery blood flow for total kidney volume, and averaged across kidneys.

Multi-parametric maps were computed using in-house software (MATLAB, The Mathworks Inc., Natick, MA, USA).

##### T*_1_* mapping

SE-EPI/bFFE IR data were fit to generate *T*_1_/apparent *T*_1_ (*T*_1_*) and M_0_ maps.

##### DWI mapping

Apparent diffusion coefficient (ADC) maps were generated from fitting the log of the exponential signal decay to all *b*-values. True diffusion (*D*), pseudo-diffusion (D*) and perfusion fraction (*f*) were fit from the intravoxel incoherent motion (IVIM) model. *D* was fit from *b*-values >200 s/mm^2^, *f* was determined from the zero intercept of this fit, and D* from a mono-exponential fit using the pre-calculated values of *D* and *f* [18].

##### ASL perfusion mapping

Perfusion-weighted images were computed, realigned and averaged to create a single perfusion-weighted (Δ*M*) map. Δ*M*, inflow, *M*_0_ and *T*_1_ maps were used in a kinetic model to calculate tissue perfusion maps [17].

##### R_2_* mapping

mFFE data were fit to form *R*_2_* maps from the log of the exponential signal decay.

##### Renal cortex and medulla definition and multi-parametric MRI estimation

A histogram of *T*_1_ values within both kidneys was used to define a *T*_1_ threshold to segment renal cortex and medulla masks. These masks were applied to each multi-parametric map generating a histogram of each MRI measure for cortex and medulla [17] to which a Gaussian curve was fit, and the mode and full-width-at-half-maximum computed. In addition, the corticomedullary difference (CMD) in each multi-parametric measure was computed.

#### Renal biopsy analysis

Renal biopsy tissue underwent standard histopathological processing. In addition, tissue blocks were stained with Picro-Sirius Red Solution (Sigma-Aldrich, Direct Red 80) for Collagens I and III. Sirius red slides were uploaded to Slidepath and analysed offline using Tissue Studio 4.0 software (Definiens, Munich, Germany), a dedicated software package for quantitative digital pathology. A region-of-interest (ROI) of cortical tissue in each slide was drawn to exclude non-interstitial structures (medulla, capsule, fat, glomeruli and arterioles). Following this, the Tissue Studio 4.0 software automatically quantified the percentage of red staining (collagen) as a measure of cortical interstitial fibrosis (IF).

### Statistical analysis

Analysis was performed using SPSS version 21 (IBM^©^) and graphs generated using Prism 6 (GraphPad Software, Inc., La Jolla, CA, USA). A Shapiro–Wilk normality test was applied to each MRI measure. Normal data are expressed as mean ± SD and non-normal as median (interquartile range, IQR). Since no significant difference in multi-parametric measures was observed between right and left kidneys (paired *t*-test) the mean of both kidneys was used in analyses, except for comparisons with histological measures, when MRI measures of the left (biopsied) kidney were used. Differences between CKD and HV groups were assessed using a paired *t*-test with Bonferroni correction for multiple comparisons; P < 0.05 was considered statistically significant. Between session repeatability of MRI measures in CKD was assessed from the intra-subject coefficient of variation (CoV) and intraclass correlation coefficient (ICC). A Pearson or Spearman correlation coefficient (normality test dependent) assessed the relationship between MRI and biochemical measures [eGFR and log(UPCR)] across both CKD and HV subjects, and for the CKD group alone. We performed a multivariable linear regression analysis in SPSS to determine the association between MRI data and biochemical measures [eGFR and log(UPCR)]. This used the stepwise selection of two MRI covariables that were biologically plausible, but not measures that yield the same resultant quantifiable measure (i.e. ASL perfusion and renal artery blood flow).

To explore the association of MRI measures with degree of IF in CKD, a range of thresholds of Sirius red fibrosis scores (20–70% in 10% increments) for defining ‘Low’ and ‘High’ IF was tested [19]. ‘High’ and ‘Low’ IF values of each MRI parameter were then computed, and a mixed factorial analysis of variance (ANOVA) was performed using each IF cut-off threshold as the within-subject factor, and fibrosis level (‘High’, ‘Low’) as the between-subject factor.

## RESULTS

**FIGURE 1:**
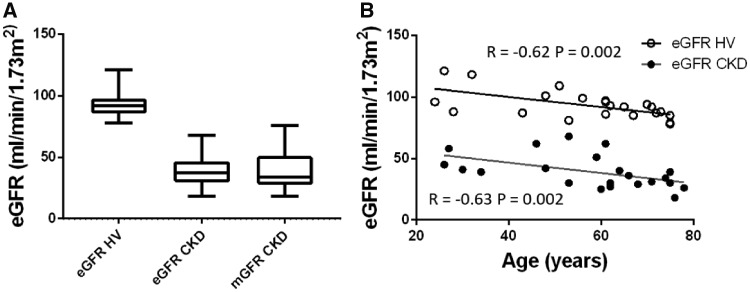
GFR. **(A)** eGFR in the HV group; eGFR and mGFR in the CKD group. **(B)** Correlation of eGFR with age, showing a significant correlation for both the HV (open circles) (*R* = −0.62, P = 0.002) and CKD (filled circles) (*R* = −0.63, P = 0.002) group.

A total of 44 participants were recruited, 22 CKD patients and age-matched HVs. Baseline characteristics are shown in Table 1 and GFR data in Figure 1A. As expected, eGFR and mGFR were highly correlated (*R* = 0.83, P < 0.001). In both HV and CKD groups, eGFR fell with age (Figure 1B). In CKD, the primary aetiology was ischaemic (*n *=* *8), interstitial (*n *=* *3) and glomerular (*n *=* *11) pathology; no patients had diabetic kidney disease. The median time from biopsy to first MRI scan was 62 (IQR 64) days.

**Table 1:** Baseline characteristics of the CKD and HV groups

Baseline demographics	CKD	HV
	(*n* = 22)	(*n* = 22)
Ethnicity (no. Caucasian)	18 (81%)	19 (86%)
Gender (no. male)	17 (77%)	17 (77%)
Age (years)	61 ± 24	61 ± 25
Height (m)	174 ± 8	176 ± 9
Weight (kg)	86 ± 12	82 ± 13
BMI (kg/m^2^)	29 ± 4	26.5 ± 3
Baseline serum creatinine (µmol/L)	159 ± 51	77 ± 11
Baseline eGFR (mL/min/1.73 m^2^)	39 ± 14	92 ± 12
Baseline mGFR (mL/min/1.73 m^2^)	34 ± 23	
UPCR (mg/mmol)	72 (IQR 108)	6 (IQR 9)
Systolic blood pressure (mmHg)	136 ± 22	130 ± 16
Diastolic blood pressure (mmHg)	85 ± 16	79 ± 13
Primary renal disease category, *n* (%)		
Glomerular disease	11 (50)	
Tubulointerstitial disease	3 (14)	
Ischaemic nephropathy	8 (36)	
Diabetes mellitus, *n* (%)	6 (27)	1 (5)
Hypertension, *n* (%)	15 (68)	5 (23)
Number on RAAS blockade, *n* (%)	13 (59)	3 (14)

BMI, body mass index; RAAS, renin–angiotensin–aldosterone system.

### Repeatability of MRI parameters in the CKD group

Table 2 provides each MRI measure for the CKD group with the associated CoV and ICC from repeatability data. CoVs were lowest for cortical and medullary *T*_1_ [both SE-EPI and bFFE readout (<4%)], cortical and medullary *R*_2_* (<7%), cortical ADC (<6%) and total kidney volume (<4%). ICCs were >0.75 for cortical *T*_1_, cortical ADC, renal artery blood flow, volume and cortical and medullary *R*_2_*.

**Table 2:** Multi-parametric MRI measures in CKD patients [shown as mean ± SD or median (IQR) dependent on normality] and assessment of their repeatability as defined by the intra-subject CoV and ICC

	Mean ± SD or	CoV (%)	ICC
	median (IQR)		
Cortical *T*_1_ SE-EPI (ms)	1574 ± 74	2.9	0.76
Medullary *T*_1_ SE-EPI (ms)	1754 ± 50	3.9	0.47
Cortical *T*_1_ bFFE (ms)	1403 ± 76	2.4	0.91
Medullary *T*_1_ bFFE (ms)	1604 ± 98	3.4	0.68
Cortical ADC (×10^−3^ mm^2^/s)	2.0 (0.2)	5.3	0.69
Medullary ADC (×10^−3^ mm^2^/s)	2.0 ± 0.2	14	0.65
Cortical *D* (×10^−3^ mm^2^/s)	1.7 ± 0.2	7.7	0.49
Medullary *D* (×10^−3^ mm^2^/s)	1.8 ± 0.2	22	0.47
Cortical *R*_2_* (/s)	20.0 ± 3.2	4.6	0.90
Medullary *R*_2_* (/s)	33.0 ± 8.0	6.8	0.91
Cortical perfusion (mL/100 g/min)	71 (50)	23	0.67
Total renal artery flow (mL/min)	490 (170)	18	0.86
Kidney volume corrected total renal artery flow (mL/100 g/min)	130 (110)	18	0.76
Total kidney volume/BSA (mL/m^2^)	170 (39)	3.8	0.94

### Differences in MRI parameters between the CKD and HV group

**FIGURE 2:**
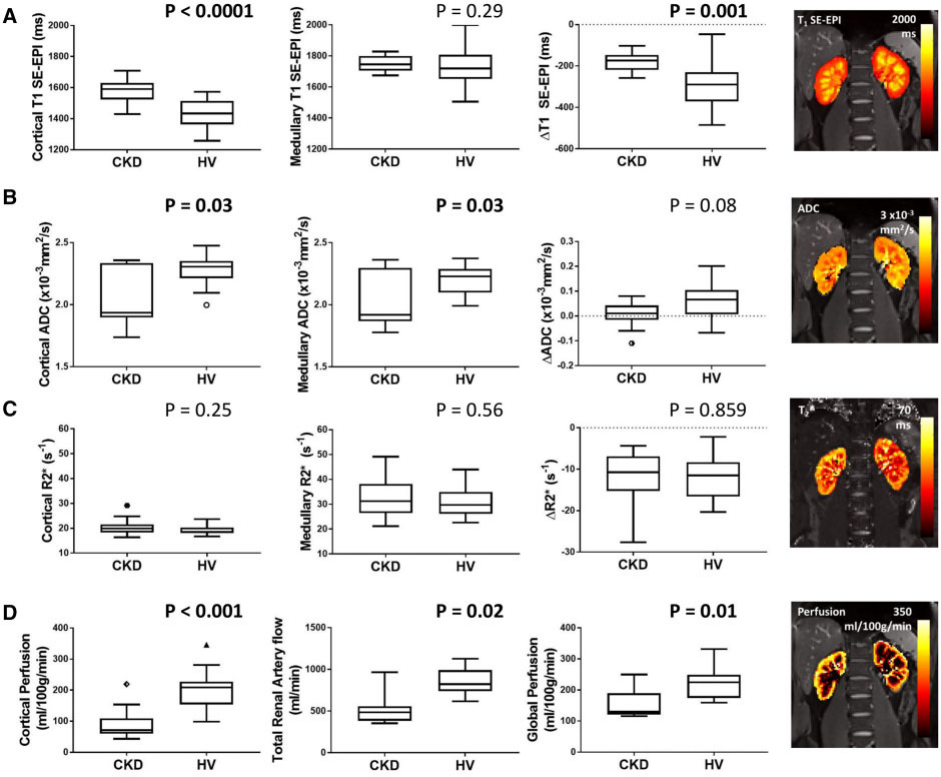
MRI parameters in the CKD and HV group for **(A)** SE-EPI *T*_1_ in cortex, medulla and CMD (Δ*T*_1_), **(B)** ADC in cortex, medulla and CMD (ΔADC), **(C)** *R*_2_* in cortex, medulla and CMD (Δ*R*_2_*), **(D)** cortical perfusion as measured by ASL, total renal artery flow and global perfusion (estimated from correcting renal artery flow for kidney volume). Significant differences are seen between the CKD and HV group for cortical *T*_1_ and Δ*T*_1_ (SE-EPI data shown here, with the correlation between SE-EPI and bFFE *T*_1_ measures shown in Supplementary data, Figure S2), cortical and medullary ADC, cortical perfusion, cortical and medullary ADC, total renal artery flow and global perfusion.

Significant differences in MRI parameters were observed between the CKD and HV group (Figure 2).

#### Renal macro- and microstructure

Kidney volume was significantly lower in the CKD group compared with HVs (CKD: 322 ± 114 mL, HV: 396 ± 99 mL; P = 0.02) as well as kidney volume corrected for BSA [CKD: 165 (39) mL/m^2^, HV: 194 ± 28 mL/m^2^; P = 0.009]. These data are shown graphically in Supplementary data, Figure S1.

Cortical SE-EPI *T*_1_ was significantly longer in the CKD group compared with HVs (CKD: 1574 ± 74 ms, HV: 1432 ± 87 ms; P < 0.001, Figure 2A), but no significant difference was evident in medullary *T*_1_ (CKD: 1754 ± 50 ms, HV: 1731 ± 134 ms; P = 0.29). Both CKD and HV groups had a significantly longer medullary *T*_1_ than cortex (P < 0.0001). Increased cortical *T*_1_ in the CKD group resulted in a significantly reduced *T*_1_ CMD (Δ*T*_1_) in the CKD group compared with HVs (CKD: −183 ± 44 ms, HV: −298 ± 98 ms; P = 0.001). This finding was replicated in *T*_1_ bFFE data (Δ*T*_1_ CKD: −201 ± 71 ms, Δ*T*_1_ HV: −327 ± 77 ms; P < 0.001), which showed a strong correlation between *T*_1_ SE-EPI and bFFE measures, Supplementary data, Figure S2A. It was also noted that both HV and CKD groups showed a trend for increased cortical *T*_1_ with age (CKD: *R* = 0.42, P = 0.07, HV: *R* = 0.42, P = 0.06).

ADC was lower in the CKD group compared with HVs in both cortex [CKD: 1.94 (0.44) × 10^−3^ mm^2^/s, HV: 2.3 ± 0.1 × 10^−3^ mm^2^/s; P = 0.030] and medulla (CKD: 2.03 ± 0.22 × 10^−3^ mm^2^/s, HV: 2.2 ± 0.1 × 10^−3^ mm^2^/s; P = 0.031; Figure 2B). ADC was significantly higher in the cortex than medulla in the HV group resulting in a positive ADC CMD (ΔADC) (P < 0.01); no significant CMD was found in the CKD group. Similar trends were found for true diffusion (*D*) (Supplementary data, Figure S2).

#### Renal oxygenation

There was no significant difference in cortical or medullary *R*_2_* between CKD and HV groups. Cortical *R*_2_* was significantly (P < 0.001) lower than medullary *R*_2_* in both CKD (cortex: 20.4 ± 3.2/s, medulla: 32.6 ± 8.0/s) and HV (cortex: 18.6 ± 2.2/s, medulla: 31.0 ± 6.1/s) groups.

#### Renal haemodynamics

Cortical perfusion assessed using ASL was significantly lower in the CKD group than the HV group [CKD: 71 (49) mL/100 g/min, HV: 200 ± 56 mL/100 g/min; P < 0.001], as was total renal artery blood flow [CKD: 486 (174) mL/min, HV: 856 ± 160 mL/min; P = 0.02] and global kidney perfusion [CKD: 132 (108) mL/100 g/min, HV: 225 ± 75 mL/100 g/min; P = 0.0078].

### Association between MRI data and biochemical measures

**FIGURE 3:**
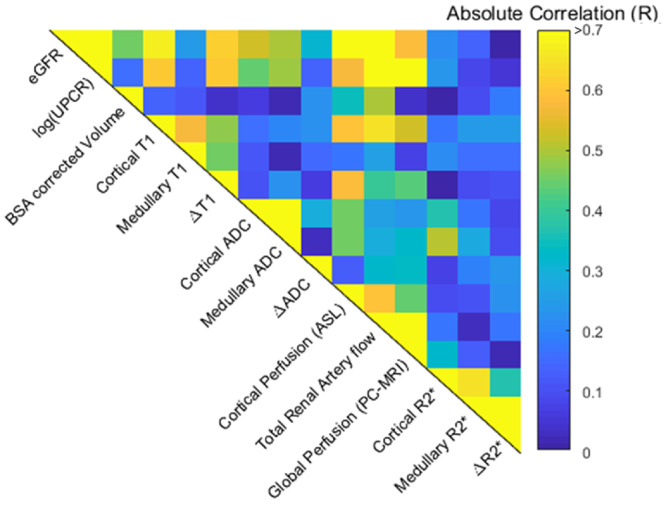
Correlation matrix for combined HV and CKD groups of biochemical measures [eGFR and log(UPCR)] and multi-parametric MRI measures. Significant correlations of MRI measures with biochemical measures are shown in Figure 4. Between the multi-parametric MRI measures, significant correlations are observed between cortical *T*_1_ and cortical perfusion (*R* = −0.595, P < 0.001), total renal artery blood flow (*R* = −0.655, P < 0.001), global perfusion (*R* = −0.435, P = 0.001); between *T*_1_ CMD (Δ*T*_1_) and cortical perfusion (*R* = −0.587, P < 0.001) and total renal artery blood flow (*R* = −0.397, P = 0.05). Correlations are also seen between cortical ADC and cortical perfusion (*R* = 0.452 P = 0.02). Between haemodynamic measures, significant correlations were observed between cortical perfusion and total renal artery flow (*R* = 0.596, P = 0.002), and cortical perfusion and global perfusion (*R* = 0.44, P = 0.04).

**FIGURE 4:**
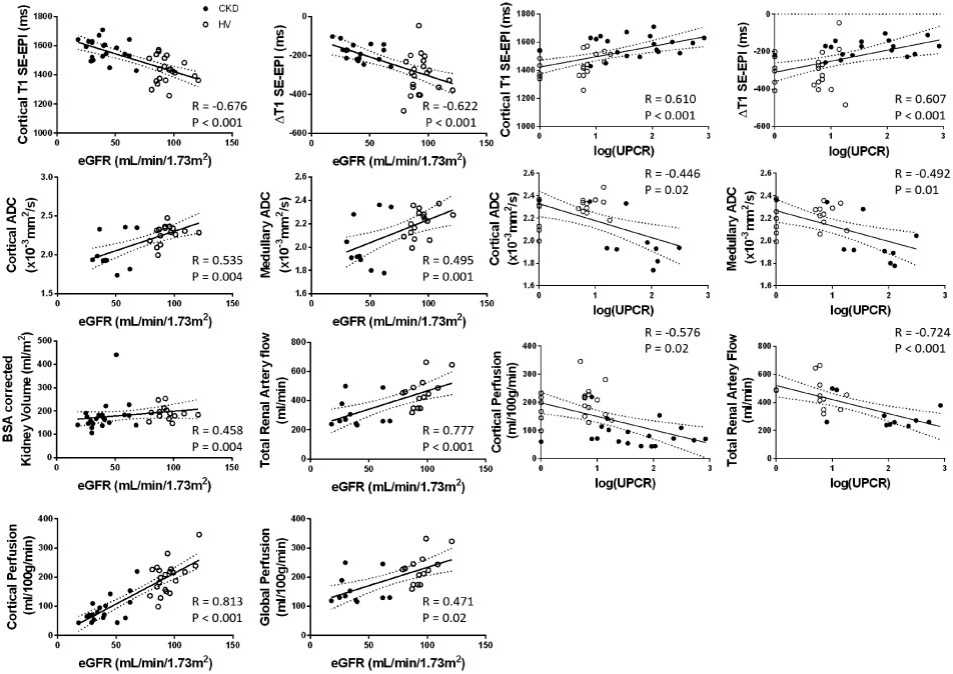
MRI measures which show significant correlations with eGFR and log(UPCR) across combined HV (open circles) and CKD (filled circles) groups.

Figure 3 shows the correlation matrix of the univariate analyses of multi-parametric MRI and biochemical measures [eGFR and log(UPCR)] for the combined CKD and HV groups, with graphs of significant correlations shown in Figure 4. Cortical *T*_1_ correlated strongly with eGFR and log(UPCR), as did *T*_1_ CMD, cortical ADC and to a lesser extent medullary ADC. ADC CMD did not correlate with either eGFR or log(UPCR). Kidney volume was positively correlated with eGFR, with a stronger correlation after BSA correction. Haemodynamic measures of cortical perfusion, total renal artery flow and global perfusion all correlated strongly with eGFR and log(UPCR). There were no significant correlations between *R*_2_* and any biochemical measures. Significant correlations were observed between some but not all MRI measures. As expected, there were significant correlations between haemodynamic measures (cortical perfusion, total renal artery flow and global perfusion), and for individual MRI parameters between cortical and medullary measures. In addition, cortical *T*_1_ correlated with perfusion, total renal artery blood flow and global perfusion; *T*_1_ CMD correlated with perfusion and total renal artery blood flow; and cortical ADC with perfusion.

Using multivariable linear regression, the best predictive model of eGFR from MRI measures included cortical perfusion and cortical *T*_1_ [eGFR = 143 – 0.77 × (cortical *T*_1_) + 0.27 × (cortical perfusion); *R* = 0.87, P < 0.001]. Cortical perfusion and cortical *T*_1_ resulted in a weaker association to predict log(UPCR) [log(UPCR) = −2.8 + 0.03 × (cortical *T*_1_) −0.03 × (cortical perfusion); *R* = 0.58, P = 0.001]. A significant association with log(UPCR) was also found for *T*_1_ CMD (Δ*T*_1_) and cortical ADC [log(UPCR) = 5.27 + 0.002 × (Δ*T*_1_) − 1.72 × (cortical ADC); *R* = 0.61, P = 0.005].

**FIGURE 5:**
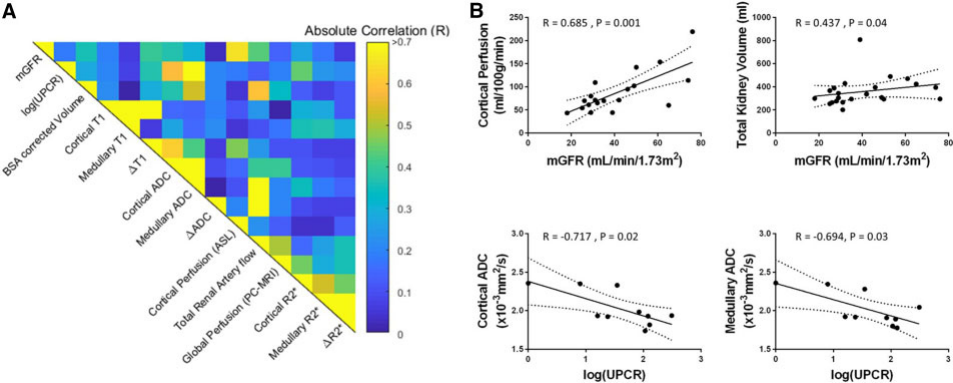
**(A)** Correlation matrix for the CKD group alone of biochemical measures [mGFR and log(UPCR)] and multi-parametric MRI measures. **(B)** MRI measures which show significant correlations with mGFR and log(UPCR) for the CKD group alone.

Figure 5A shows the correlation matrix of MRI measures and biochemical measures [mGFR and log(UPCR)] for the CKD group alone. There was a significant correlation of mGFR with cortical perfusion and mGFR with total kidney volume [Figure 5B, though this was not significant for BSA corrected kidney volume (Figure 5A)]. Log(UPCR) had a negative correlation with cortical and medullary ADC. Using multiple linear regression, mGFR was predicted by combining cortical perfusion and cortical *T*_1_ [mGFR = −38.2 + 0.035 × (cortical *T*_1_) + 0.296 × (cortical perfusion); *R* = 0.73, P = 0.03], or total BSA corrected kidney volume and cortical perfusion [mGFR = 4.65 + 0.064 × (BSA corrected kidney volume) + 0.290 × (cortical perfusion); *R* = 0.76, P = 0.001]. Log(UPCR) was not significantly associated with *T*_1_ CMD and cortical ADC for the CKD group alone (*R* = 0.75, P = 0.08).

### Association between MRI parameters and histological measures

**FIGURE 6:**
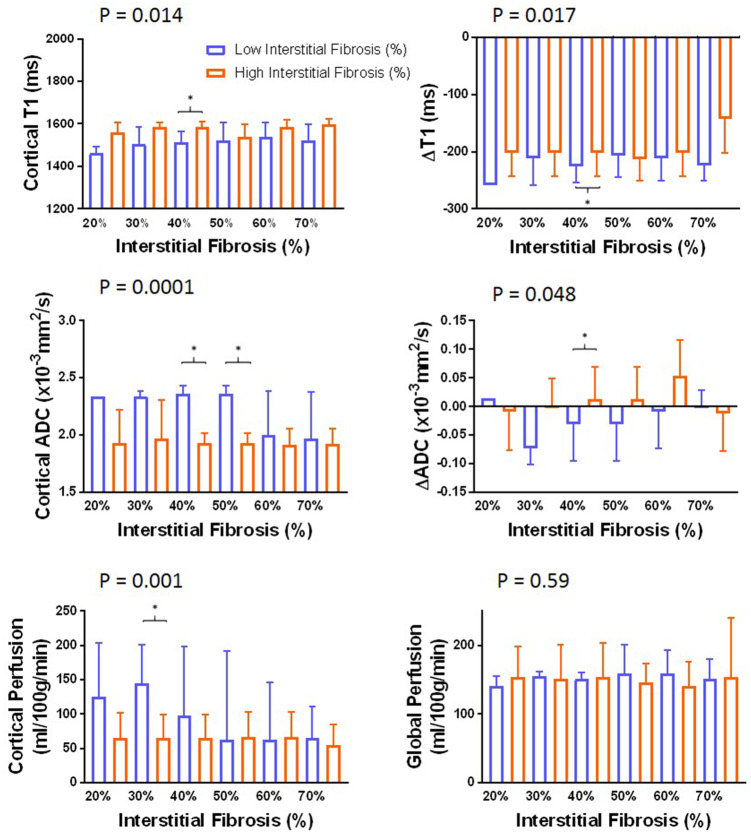
Fibrosis percentage binned into a binary factor ‘Low’ (blue) or ‘High’ (orange) IF based on varying the fibrosis threshold between 20% and 70%. It can be seen that changing the IF threshold results in a significant difference between ‘Low’ and ‘High’ IF groups for cortical *T*_1_ (P = 0.014) and *T*_1_ CMD (Δ*T*_1_) (P = 0.017), cortical ADC (P < 0.0001) and ADC CMD (ΔADC) (P = 0.048), and ASL-derived cortical perfusion (P = 0.001) as determined by ANOVA. Significant differences (Wilcoxon P < 0.05) between ‘Low’ and ‘High’ IF groups for a given IF threshold are shown by *.

Histology of each individual in the CKD group is presented in Supplementary data, Table S1. Figure 6 shows MRI parameters for ‘Low’ and ‘High’ IF groups computed for fibrosis varying thresholds between 20% and 70% in 10% increments. ANOVA revealed significant differences between ‘Low’ and ‘High’ IF groups for cortical *T*_1_, *T*_1_ CMD, cortical ADC, ADC CMD and ASL cortical perfusion. Individual comparisons showed significant differences between ‘Low’ and ‘High’ IF at 40% IF threshold for cortical *T*_1_, *T*_1_ CMD, cortical ADC and ADC CMD, with additional significant differences in cortical ADC at 50% and cortical perfusion at 30% IF thresholds.

## DISCUSSION

MRI shows promise as a non-invasive technique for evaluating whole kidney structure and function but progress towards clinical application has been slow, in part because MRI measures have generally been applied in isolation (e.g. DWI [20], BOLD [15, 21–23] and ASL [24]). We show that multi-parametric MRI can be conducted in a single scan session [25] with excellent reproducibility in HVs and persons with CKD G3–4. Several MRI parameters were able to distinguish CKD from healthy kidneys, and correlated with measures of whole kidney function (GFR and UPCR) as well as histopathological measures of IF.

The first aim of this study was to establish repeatability of multi-parametric MRI measures in a CKD group before wider application. We show individual MRI measures are highly repeatable, comparable to those previously reported in HVs by our group [17]. Our description of how the individual MRI measures are related is also important. Correlation of certain MRI measures (e.g. ASL perfusion and renal artery blood flow, Figure 3) confirms technical validity. Conversely, the observation that a number of MRI measures do not correlate confirms MRI measures are sensitive to different structural or functional alterations, supporting multi-parametric MRI.

We report significant differences in renal microstructure between the CKD and HV group assessed by *T*_1_ mapping and DWI. Cortical *T*_1_ was increased in CKD, resulting in a significantly reduced *T*_1_ CMD. Increased *T*_1_ reflects increased extracellular fluid resulting from inflammation, interstitial oedema or cellular swelling [18], or fibrosis due to the association of collagen with supersaturated hydrogel [19, 26]. In tandem, the CKD group had lower cortical ADC and *D*-values. Reduced diffusion of water molecules may be due to fibrosis [19, 27], with the accumulation of cells including fibroblasts in the interstitial space during renal fibrogenesis and collagen deposition restricting water diffusion. Along with microstructural changes, we observed a significant reduction in kidney volume in CKD compared with HVs.

Haemodynamic assessment of the kidney included ASL perfusion mapping and PC-MRI renal artery blood flow, which when corrected for kidney volume estimates global renal perfusion (with the caveat that blood flow is equally distributed across kidney volume, thus not accounting for lower perfusion in the medulla or fibrotic tissue). Consistent with previous studies, total renal artery blood flow [28], cortical perfusion [24, 29–31] and global perfusion were reduced in CKD compared with HVs, likely reflecting vascular rarefaction in chronic fibrosis. Our measures of cortical perfusion may appear lower than other published values [24, 29–31], which may reflect a higher proportion of ischaemic nephropathy in our cohort, or the higher proportion of patients with lower GFR.

We observed no meaningful differences in cortical or medullary *R*_2_* values between CKD and HVs, noting our values show good agreement with previous studies [23]. Current data regarding renal *R*_2_* data are conflicting; studies report both increased cortical *R*_2_* in CKD compared with HVs attributed to lower renal tissue oxygenation [32–34], and no difference [23]. Some of this variability may be explained in part by technical factors (shimming method, ROI placement), although Pruijm *et al.* [35] recently showed BOLD *R*_2_* correlates with a faster decline in eGFR and a higher risk of adverse renal outcomes.

As expected, MRI measures that differed significantly between CKD and HV groups strongly correlated with GFR (cortical *T*_1_, cortical and medullary ADC, cortical perfusion, BSA corrected kidney volume, renal artery flow and global perfusion). Within the CKD group (smaller range of renal function and sample size), haemodynamic and structural measures (cortical perfusion, cortical and medullary ADC, and kidney volume) correlated with mGFR. We also observed correlations between MRI measures and proteinuria. Cortical *T*_1_ and *T*_1_ CMD had a strong positive correlation with log(UPCR), coupled with a negative correlate of cortical and medullary ADC with log(UPCR). ASL perfusion, renal artery blood flow and global perfusion negatively correlated with log(UPCR). Mao *et al.* [20] reported a negative correlation between diffusion measures and proteinuria and a positive correlation with eGFR. There are few reports relating proteinuria and other MRI measures. An important observation is that MRI measures retained independent associations with eGFR (cortical perfusion and cortical *T*_1_) and proteinuria [*T*_1_ CMD (Δ*T*_1_) and cortical ADC], supporting the use of multi-parametric MRI.

A number of previous MRI studies have attempted to non-invasively measure renal fibrosis [36], with one or two MRI measures being compared with subjective or objective measures of IF from biopsy. Mao *et al.* [20] compared diffusion measures with subjective assessment of renal fibrosis in CKD Stage G1–5 and showed a negative correlation between diffusion measures and fibrosis score. In renal transplant recipients, Friedli *et al.* [19] reported reduced ADC CMD to be the best predictor of ‘High’ IF defined to be >40% IF. Our results are broadly consistent, with ADC and ADC CMD being significantly different between ‘Low’ and ‘High’ IF, and most apparent at 40% IF. Additionally, we show *T*_1_ cortex, *T*_1_ CMD difference and ASL cortical perfusion are predictors of IF. Our sample size renders these results exploratory, but they support the potential of combining multi-parametric MRI measures to improve prediction of IF, clinically important as IF is one of the strongest predictors of CKD progression.

This study has limitations, with a relatively small sample size and a range of disease types and age span, though the latter was addressed by including a paired age-matched HV group.

In conclusion, multi-parametric MRI measures have good reproducibility in CKD, can distinguish CKD from healthy kidneys, and correlate with biochemical and histopathological measures. Further evaluation requires multicentre studies to assess MRI measures across all CKD stages and in larger numbers to identify the optimal combination of MRI measures, and longitudinal studies to assess the prognostic value of multi-parametric MRI compared with currently available biochemical measures.

## Supplementary Material

gfz129_Supplementary_Data
