# Supplementary material for: Quantitative assessment of renal structural and functional changes in chronic kidney disease using multi-parametric magnetic resonance imaging
Source: Nephrol Dial Transplant. 2019 Jun 29;35(6):955–64. doi: 10.1093/ndt/gfz129 (PMC7282828; doi:10.1093/ndt/gfz129)
Supplement: gfz129_Supplementary_Data [file gfz129_supplementary_data.zip › gfz129_New_Supplementary_Figure/Supplementary table.docx]

**Table S1**: Histological data in the Chronic Kidney Disease (CKD) group. IgA, immunoglobulin A; FSGS, focal segmental glomerulosclerosis

| CKD Participant | Primary renal Disease | Interstitial fibrosis  (%) |
| --- | --- | --- |
| 1 | IgA Nephropathy | 81 |
| 2 | Interstitial Fibrosis | 38 |
| 3 | Ischaemic Nephropathy | 30 |
| 4 | IgA Nephropathy | 50 |
| 5 | Oligomeganephronia | 16 |
| 6 | Ischaemic Nephropathy | 48 |
| 7 | Ischaemic Nephropathy | 60 |
| 8 | Immune Complex Glomerulonephritis (type unspecified) | 64 |
| 9 | IgA Nephropathy | 60 |
| 10 | IgA Nephropathy | 35 |
| 11 | IgA Nephropathy | 57 |
| 12 | Secondary FSGS (Chronic Pyelonephritis) | 68 |
| 13 | Mesangio-Proliferative Glomerulonephritis Type 1 | 23 |
| 14 | Ischaemic Nephropathy | 18 |
| 15 | Ischaemic Nephropathy | 80 |
| 16 | Tubulointerstitial Nephritis | 61 |
| 17 | Ischaemic Nephropathy | 76 |
| 18 | Ischaemic Nephropathy | 40 |
| 19 | Accelerated Hypertension | 53 |
| 20 | IgA Nephropathy | 77 |
| 21 | Tubulointerstitial Nephritis | 53 |
| 22 | Membranous Glomerulonephritis | 49 |
| **Mean±SD** |  | **53±20** |
