# Supplementary figures and images for: Quantitative assessment of renal structural and functional changes in chronic kidney disease using multi-parametric magnetic resonance imaging
Source: Nephrol Dial Transplant. 2019 Jun 29;35(6):955–64. doi: 10.1093/ndt/gfz129 (PMC7282828; doi:10.1093/ndt/gfz129)

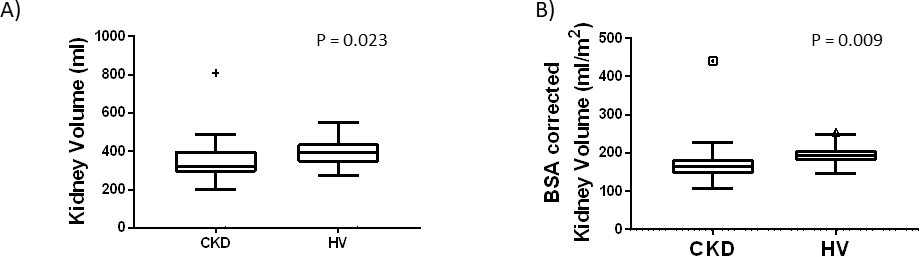

Supplement: gfz129_Supplementary_Data [file gfz129_supplementary_data.zip › gfz129_New_Supplementary_Figure/Suppfig1.TIF]

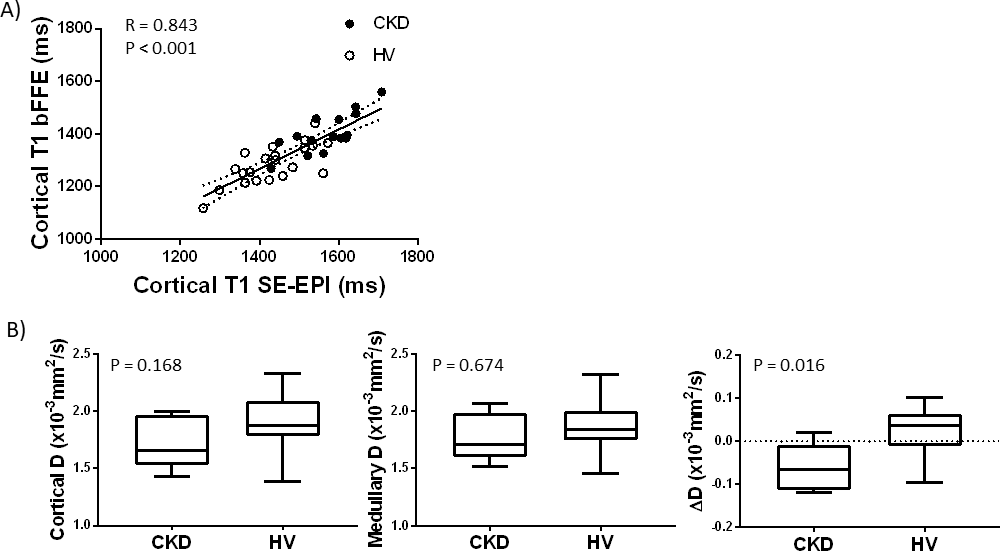

Supplement: gfz129_Supplementary_Data [file gfz129_supplementary_data.zip › gfz129_New_Supplementary_Figure/Suppfig2.TIF]
